# Ablation of prion protein immunoreactivity by heating in saturated calcium hydroxide

**DOI:** 10.1186/1756-0500-1-99

**Published:** 2008-10-28

**Authors:** Justin J Greenlee, Eric M Nicholson, Amir N Hamir, Gary P Noyes, Mark T Holtzapple, Marcus E Kehrli

**Affiliations:** 1 Virus and Prion Diseases of Livestock Research Unit, National Animal Disease Center, USDA, Agricultural Research Service, 2300 Dayton Avenue, Ames, IA, USA, 50010; 2 Oceaneering Space Systems, Houston, TX, USA 77058; 3 Department of Chemical Engineering, Texas A&M University, College Station, TX, USA 77843

## Abstract

**Background:**

Prions, the infectious agents that cause transmissible spongiform encephalopathies (TSEs), are relatively resistant to destruction by physical, enzymatic, and chemical treatments. Hydrolysis in boiling saturated calcium hydroxide (limewater) utilizes inexpensive chemicals to digest protein components of offal. The purpose of this work was to determine if incubating brain material from scrapie-infected sheep in near-boiling saturated calcium hydroxide solution (Ca(OH)_2_) would abolish immunoreactivity of the infectious prion (PrP^Sc^) as determined by western blot.

**Findings:**

After incubating for as few as 10 minutes in saturated calcium hydroxide at 99°C, immunoreactivity of protease resistant bands by western blot analysis is completely lost.

**Conclusion:**

Boiling in limewater may offer an alternative for disposal of carcasses and enable alternative uses for rendered products from potentially infected carcasses.

## Background

Scrapie in sheep, bovine spongiform encephalopathy (BSE), chronic wasting disease (CWD) of deer and elk, and Creutzfeldt-Jakob disease in humans are chronic neurodegenerative diseases associated with the accumulation of the protease-resistant, disease-associated isoform of the prion protein (PrP^Sc^) in selected regions of the central nervous system. PrP^Sc ^is relatively resistant to inactivation by standard decontamination procedures [1] and can remain infectious after undergoing sterilization procedures under high pressure, treatment with disinfectants, or exposure to dry heat.[2]

The host of any particular prion disease determines the potential distribution of PrP^Sc ^in the tissues. Brain, spinal cord, and distal ileum and in some species, peripheral lymphoid tissues contain infectious material (PrP^Sc^). Removal, handling, and disposal of tissue with the potential to harbor infectious PrP^Sc ^represent a challenge to the meatpacking and by-product industries. Efficient methods of inactivating potentially infectious material are needed.

A number of procedures have been described to decontaminate surfaces or reusable medical devices, [3-7] but few of these methods are appropriate for decontamination of large volumes of animal tissue from TSE-infected animals. Chemical means of inactivation such as concentrated hypochlorite (bleach) solutions or sodium hydroxide solution can damage equipment, cause respiratory irritation in workers exposed to fumes, pose problems with disposal, or may not be appropriate for processing large amounts of material. Disposal of large volumes of chemically treated material may present problems for some municipal sewage systems. High-pressure and temperature methods of inactivation [3] or reducing infectivity[8] of tissues and proteolytic digestion by enzymes secreted by thermophylic bacteria[9] have not been demonstrated to be effective on a large scale.

Methods for detecting abnormal prion in meat and bone meal (MBM) are under development,[10] but methods that inactivate abnormal prions and leave material with by-product value (concentrated amino acids, feed additive, fertilizer, etc.) may be more desirable. Incineration can inactivate the agent,[2] but precludes any recovery or reuse of raw material.

Inexpensive, effective means of inactivating prions in offal are needed for safe disposal of carcasses and to ensure the safety of animal by-products if used as a feed source. Ultimately, the success of any potential method of inactivation must be measured by comparing infectivity in cell cultures[11] or animal models of prion disease, but western blot can be used to screen for unfolding or denaturation of PrP^Sc^, which would result in a loss of reactivity with prion-specific antibodies. The purpose of this study was to determine if incubating brain material from scrapie-infected sheep in near-boiling saturated calcium hydroxide solution would abolish immunoreactivity of the infectious prion (PrP^Sc^) as determined by western blot.

## Methods

### Preparation of brain material

Brain immunoreactive for PrP^Sc ^by immunohistochemistry and western blot from intracerebrally inoculated sheep or brain from scrapie-free control animals[12] was homogenized at a final concentration of 40% (w/v) in 10 mM Tris 5 mM MgCl_2_, pH 7.5 using a tissue homogenizer (Powergen 125 homogenizer, Fisher Scientific) with disposable probe. Secondly, ultrasonic dismembranation (Fisher Scientific Model 500 Ultrasonic Dismembrator, Fisher Scientific) was performed for four 30-second intervals in an ice bath with brief vortex mixing between sonication. The samples were then centrifuged at 2,000 × g for 2 minutes at room temperature. The supernatant was then treated with 100 U/mL benzonase (Novagen) at 37°C for 1 hour. The material was used immediately for the inactivation experiments or frozen at -80°C for later use.

#### Calcium Hydroxide (Ca(OH)_2 _treatment

The 40% brain homogenates were diluted to a final concentration of 10% (w/v) brain in saturated Ca(OH)_2 _containing excess insoluble Ca(OH)_2_. Samples of 10% (w/v) brain homogenate in 10 mM Tris 5 mM MgCl_2_, pH 7.5 were processed in parallel to those homogenized in saturated Ca(OH)_2 _to serve as a heat-only control. All samples except from the zero timepoint were placed at 99°C for incubation. The zero timepoint sample and samples measured at subsequent timepoints were neutralized by a 1:6.9 dilution into 1 M Tris-HCl to stop the reaction. Ice-cold acetone (4 volumes) was added to each sample, and samples were then placed at -20°C for at least 1 hour.

### Preparation of samples and analysis by western blot

Following incubation at -20°C for 1 hour, the calcium hydroxide treated samples were centrifuged at 13,000 × g for 10 minutes at room temperature. The supernatant was discarded and the protein pellet was allowed to air dry. Once dry, the pellet was dissolved in SDS-PAGE sample buffer such that 1.2 mg tissue equivalent (assuming 100% yield) was loaded on the gel. Positive and negative scrapie control samples were homogenized in 10 mM Tris-HCl 5 mM MgCl_2 _pH 7.5 and loaded on a gel at ~1 mg tissues equivalent in SDS-PAGE sample buffer without treatment or after 240 minutes at 99°C. For the control samples, proteinase K (USB) was added to a final concentration of 80 μg/ml; digestion was conducted at 37°C for 1 hour and stopped by the addition of Pefabloc (Roche Diagnostics) to a final concentration of 0.1 mg/mL.

For SDS-PAGE and western blot, a 4–20% commercially prepared SDS-PAGE gel was loaded and run according to the manufacturer's instructions, blotted to a polyvinylidene difluoride (PVDF) membrane (GE Healthcare) and blocked with 3% bovine serum albumin. Western blot detection was conducted using mouse anti-PrP monoclonal antibody P4 (R-Biopharm AG), which targets to amino acids 89–104 of the ovine prion protein sequence,[13] at a 1:10,000 dilution (0.1 μg/mL) as the primary antibody. Western blots were repeated using monoclonal antibody F99/97.6.1, which targets residues 220–225 of ovine prion protein sequence.[14] A biotinylated sheep anti-mouse secondary antibody at 0.05 μg/ml and a streptavidin-horseradish peroxidase (HRP) conjugate (GE Healthcare) were used according to the manufacturer's instructions in conjunction with the ECL Plus detection system (GE Healthcare) and imaged using a Kodak Image Station In-Vivo F. Primary antibody incubations were conducted with the membrane at either room temperature for 1 hour or 4°C overnight (≥ 12 hours). Secondary antibody and streptavidin-HRP conjugate incubations were conducted at room temperature for 1 hour. Band intensity was determined using Kodak Molecular Imaging software.

## Results and discussion

**Figure 1:**
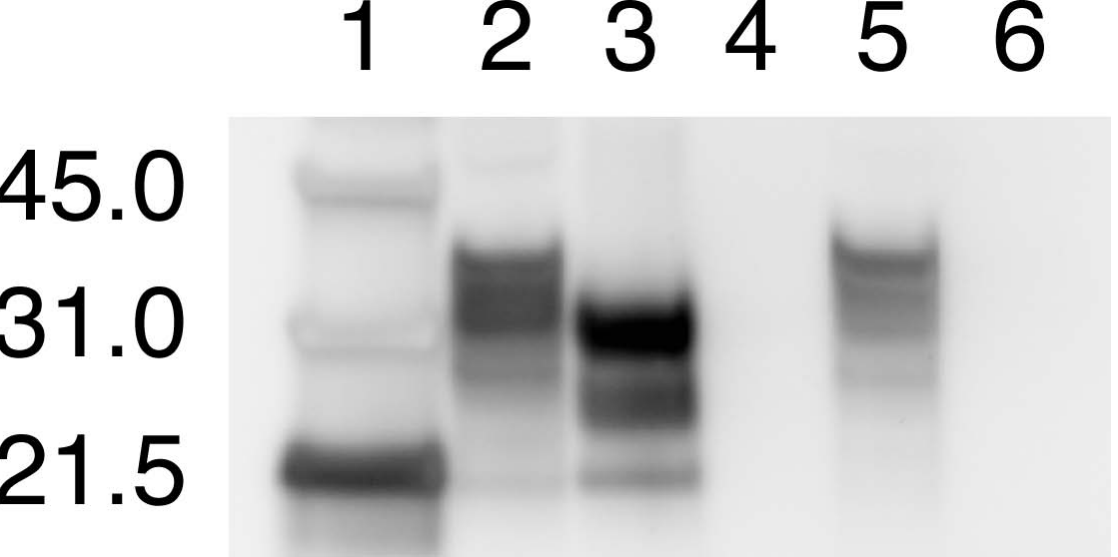
**Western blot of non-treated brain homogenates**. Representative western blots of scrapie-positive and scrapie-negative sheep brain homogenates. Lane 1-Molecular weight marker; lane 2-Scrapie-positive brain without proteinase K digestion; lane 3-Scrapie-positive brain treated with proteinase K; lane 4-Blank; lane 5-Scrapie-negative brain without proteinase K digestion; lane 6-scrapie-negative brain with proteinase K digestion. Only the scrapie-positive brain homogenate exhibits proteinase K resistance.

**Figure 2:**
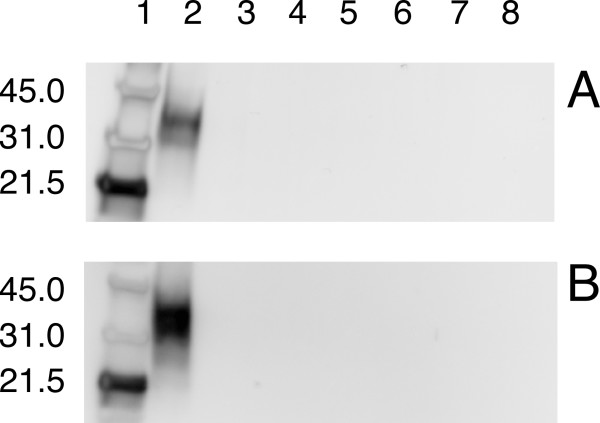
**Western blot after calcium hydroxide treatment**. Western blot analysis of calcium hydroxide treatment of scrapie-negative (A) and scrapie-positive (B) sheep brain homogenate. For both, Lane 1-molecular weight marker; lane 2–0 minutes; lane 3–10 minutes; lane 4–20 minutes; lane 5–30 minutes; lane 6–60 minutes; lane 7–120 minutes; lane 8–240 minutes. PrP immunoreactivity is abolished in scrapie-positive samples after 10 minutes treatment with calcium hydroxide.

Scrapie-positive and scrapie-negative samples were confirmed based upon the presence or absence, respectively, of proteinase K-resistant PrP (Figure 1). For both scrapie-positive and scrapie-negative samples, PrP is clearly visible in the first sample taken after dilution into saturated Ca(OH)_2_, however, after only 10 minutes there is virtually no detectable PrP in either the scrapie-positive or scrapie-negative samples (Figure 2) on a western blot. Analysis of the western blot indicates that greater than 95% of all detectable PrP is hydrolyzed in the first 10 minutes. Samples homogenized in 10 mM Tris-HCl 5 mM MgCl_2 _pH 7.5 remained immunoreactive after 240 minutes at 99°C (results not shown).

As the infectious agent, reduction in PrP^Sc ^levels is strongly suggestive of reduced infectivity. However, PrP^Sc ^is detected indirectly *via *western blot and disruption of antibody-PrP^Sc^interactions for which there would be no corresponding reduction in infectivity could be one potential explanation for lack of immunoreactivity. Western blots were performed with multiple antibodies with different binding epitopes to assure that lack of immunoreactivity was not due to loss of an epitope at a single site. Failure to reduce PrP^Sc ^levels while reducing infectivity is much less likely.[15]

Base-catalyzed hydrolysis using sodium hydroxide at concentrations ≥ 1 N is an accepted means of decontamination of PrP^Sc^-contaminated material.[16] Reports indicate that even this fails to completely inactivate some PrP^Sc ^isolates.[17] Failures may be more a result of the consumption of hydroxide ion in the hydrolysis than an inability of 2N NaOH to hydrolyze PrP^Sc^. The pH of 2 N NaOH is greater than 14. In contrast, saturated Ca(OH)_2 _is nearer pH 12.5, which is still sufficient to rapidly hydrolyze peptide bonds. As the hydroxide ions are consumed in the case of NaOH, the pH of the solution would drop; however, using a slurry of saturated Ca(OH)_2 _solution and insoluble Ca(OH)_2 _the hydroxide ion concentration and thus the pH is readily maintained. Additionally, Na^+ ^ions are poorly tolerated in feed, whereas Ca^2+ ^ions are much more readily tolerated. Thus, although Ca(OH)_2_, is a weaker base than the often-used NaOH it offers some practical advantages to prion inactivation on an industrial scale for the purposes of producing a safe, alternative by-product.

New methods of prion inactivation for application to offal would reduce complications with carcass disposal and assure safety of rendered materials used as an animal feed source. Immunoassay alone is not sufficient to evaluate the efficacy of a particular method of inactivation [11] as removal of all immunoreactive PrP^Sc ^correlates poorly with levels of infectivity measured by bioassay. Animal inoculations appear to be a standardizable method for the evaluation of prion inactivation, but a 15-month incubation period is required to guarantee total absence of infectivity.[3] Results of this study demonstrate the potential for heating in near-boiling saturated Ca(OH)_2 _as a method of PrP^Sc ^inactivation with practical and safety advantages to the handling of the hydrolysate. However, confirmation of loss of infectivity requires bioassay or cell culture infectivity studies as loss of immunoreactivity in WB is not equivalent to loss of infectivity. Bioassay using inoculum treated with heating in saturated calcium hydroxide is currently underway. Because strain differences to some methods of inactivation have been demonstrated,[6] it may be necessary to pursue inactivation studies using material derived from cattle with BSE and cervids with CWD.

## Competing interests

Gary Noyes and Mark Holtzapple are named as co-inventors on a U.S. Patent Application of the boiling-saturated-calcium-hydroxide protein-depolymerization process. Neither has any stock or ownership in the company that has licensed the patent from Texas A&M University.

## Authors' contributions

JG, EN, MH, GN, and MK conceived of the idea for this research. JG and EN designed the experiments, analyzed and interpreted data, and wrote the manuscript. AH provided tissues and reagents and critically reviewed the manuscript. MK supervised data collection and reviewed the manuscript. All the authors have read and approved the final manuscript.
